# Alternative and Unconventional Feeds in Dairy Diets and Their Effect on Fatty Acid Profile and Health Properties of Milk Fat

**DOI:** 10.3390/ani11061817

**Published:** 2021-06-18

**Authors:** Sylvie Hadrová, Kateřina Sedláková, Ludmila Křížová, Svetlana Malyugina

**Affiliations:** 1 Agrovýzkum Rapotín Ltd., 78813 Vikýřovice, Czech Republic; sylvie.hadrova@vuchs.cz (S.H.); svetlana.malyugina@vuchs.cz (S.M.); 2 Department of Animal Breeding, Animal Nutrition and Biochemistry, Faculty of Veterinary Hygiene and Ecology, University of Veterinary Sciences Brno, 61242 Brno, Czech Republic; sedlakovak@vfu.cz

**Keywords:** dairy cows, health, milk fat quality, indices, algae, okara, camelina, herbs and spices, tannins

## Abstract

**Simple Summary:**

Milk fat is an important compound in human nutrition. From a nutritional point of view, the production of milk with a higher content of polyunsaturated fatty acids, especially of those from the n3 group, is desirable because consumption of a diet with a lower n6/n3 ratio is considered to be beneficial for humans. The most effective way to achieve this goal is via dietary manipulations in ruminants. In addition to the feedstuffs commonly used in dairy animal nutrition, there are some alternative or unconventional feedstuffs that are often used for other purposes, e.g., for the reduction of methane production in the rumen. However, such feedstuffs can also alter the fatty acid profile of milk, and thus they can have an impact on the health properties of milk fat.

**Abstract:**

Milk fat is an important nutritional compound in the human diet. From the health point of view, some fatty acids (FAs), particularly long-chain PUFAs such as EPA and DHA, have been at the forefront of interest due to their antibacterial, antiviral, anti-inflammatory, and anti-tumor properties, which play a positive role in the prevention of cardiovascular diseases (CVD), as well as linoleic and γ-linolenic acids, which play an important role in CVD treatment as essential components of phospholipids in the mitochondria of cell membranes. Thus, the modification of the FA profile—especially an increase in the concentration of polyunsaturated FAs and n-3 FAs in bovine milk fat—is desirable. The most effective way to achieve this goal is via dietary manipulations. The effects of various strategies in dairy nutrition have been thoroughly investigated; however, there are some alternative or unconventional feedstuffs that are often used for purposes other than basic feeding or modifying the fatty acid profiles of milk, such as tanniferous plants, herbs and spices, and algae. The use of these foods in dairy diets and their effects on milk fatty acid profile are reviewed in this article. The contents of selected individual FAs (atherogenic, rumenic, linoleic, α-linolenic, eicosapentaenoic, and docosahexaenoic acids) and their combinations; the contents of n3 and n6 FAs; n6/n3 ratios; and atherogenic, health-promoting and S/P indices were used as criteria for assessing the effect of these feeds on the health properties of milk fat.

## 1. Introduction

An increased demand for milk and dairy products (especially milk and butter) has become a worldwide trend in recent years [1,2]. Fatty acids (FAs) in milk fat are considered to be important nutritional compounds in the human diet [3]. From a nutritional point of view, the production of milk with a higher proportion of polyunsaturated fatty acids (PUFAs), especially those from the n3 group, is desirable because diets with a higher content of n3 and a lower content of n6 FAs—that is, with a lower n6/n3 ratio—are considered to be healthier for humans [4,5]. Considering the importance of PUFAs in human health and nutrition [6], the modification of the FA profile of milk fat has been a target of many studies. There are many factors influencing the FA profile of milk, with nutrition being the crucial one [3]. Major dietary factors, such as the type and amounts of forages or concentrates in the diet, the forage/concentrate ratio, and the supplementation of diets with fats or oil supplements, have been widely studied (reviewed recently in [3]). However, there are also some alternative or unconventional feedstuffs that are often used for different purposes (e.g., for the reduction of methane emissions or for buffering rumen pH) that can affect the FA profile of milk fat. Thus, this review evaluates some of those feedstuffs from the point of view of the modern demands in relation to the health characteristics of milk fat.

## 2. Milk Fatty Acids and Indices Used for the Evaluation of Milk Fat Quality

The composition of a ruminant’s diet is the main factor that can cause changes in milk FAs [3]; thus, targeted modification of the diets of ruminants can be used for the production of milk with a desirable fatty acid profile [3,7] that is in accordance with the recommendations for human nutrition [7,8]. Generally, from the health point of view, it is desirable to increase the concentration of n3 FAs in milk and dairy products and to reduce the content of certain saturated fatty acids (SFAs)—C12:0 (lauric acid), C14:0 (myristic acid), and C16:0 (palmitic acid)—as they are related to an increased risk of atherosclerosis [9].

The main n3 PUFAs are α-linolenic acid (C18:3n3; ALA), eicosapentaenoic acid (EPA, C20:5n3), docosahexaenoic acid (DHA, C22:6n3), and the less-recognized docosapentaenoic acid (DPA, C22:5n3) [10,11]. However, it should be noted that DPA also exists in the form of n6 isomer and in this form it is produced by, e.g., marine microalgae from *Schizochytrium* spp. [12]. An essential ALA is a precursor for the production of n3 long-chain (LC) PUFAs in human nutrition [10,13]. ALA is present in some seeds (e.g., flax) and green leafy vegetables [14]. The richest source of dietary EPA and DHA are fish (especially fatty fish) and marine algae [11,15]. EPA and DHA can be synthesized from the precursor by elongase and desaturase enzymes; however, in humans, they are not synthesized efficiently because the conversion rate is very low [15,16]. Some studies suggest that the conversion rate from ALA to EPA is approximately 5–8%, and that <0.5–4% of ALA is converted to DHA [17,18,19]. Therefore, they are essential for human nutrition and must be obtained via the diet [18]. Both these bioactive FAs are known to provide various health benefits. DHA has an important function in brain and nervous system development, in the process of vision, and in preventing inflammation [15,19,20]. DHA is important in the development of premature babies and small children [19], but the DHA intake is very important for adults as well. Breast milk is one of the natural sources of DHA [10]. EPA and DHA have a wide range of physiological roles linked to health benefits; thus, diets rich in fish and fish oils are related to a reduced risk of cardiovascular diseases and provide further health benefits, such as anti-carcinogenic and anti-inflammatory activity [11,21,22,23]. Even supplementation with both of these FAs seems to have a possible beneficial effect in the treatment of COVID-19 to prevent the occurrence of a “cytokine storm” [24]. As mentioned above, n3 long-chain PUFAs play a role in anti-inflammatory processes. From interleukins (a group of cytokines), interleukin-6 and interleukin-1β are suspected to play a central role in cytokine storms. In addition, these cytokines can be affected by dietary EPA and DHA intake [24]. According to [10], dietary recommendations for EPA and DHA are between 250 and 500 mg/day for adults. According to one review [25], the recommended intake of n3 LC PUFAs across health organizations is about 500 mg/day for primary prevention of cardiovascular disease.

The n6 FAs are represented by linoleic acid (LA), which is found in the seeds of most plants [14] or in vegetable oils (reviewed by [5]). LA is also an essential FA because it cannot be synthesized by humans; respectively, humans can convert only small portions of FAs (such as LA to AA) to more than 20-carbon PUFAs [14,26]; the conversion of ingested ALA to EPA and further to DHA is not a reliable source of n-3 PUFAs [25] as the conversion efficiency is generally low [26]. Gamma-linolenic acid (GLA; C18:3n6) is a representative of the n6 FAs, and in the n-6 pathway it is biosynthesized from LA. GLA can be found in human milk and in some vegetable oils such as borage (about 21% GLA), blackcurrant (about 17% GLA), and evening primrose oils (about 9% GLA) [24]. Arachidonic acid (C20:4n6), on the other hand, can be obtained primarily from foods of animal origin, such as meat, poultry, and eggs [27].

Particular health promoting effects have also been observed in rumenic acid (C18:2c9t11; RA, n7)—one of the notably beneficial conjugated linoleic acid (CLA) isomers produced during ruminal biohydrogenation [28]. CLA occurs naturally in foods derived from ruminants, and RA is the predominant CLA isomer in dairy products. CLA may provide various potential health benefits, such as antiatherogenic, anticarcinogenic, and antidiabetic effects, and moreover, it can also reduce body fat (e.g., [29]).

Milk is an important energy source, containing numerous essential nutrients, such as protein, lipids, lactose, vitamins, and minerals [30]. Milk also contains various physiologically active compounds, such as nutritionally desirable FAs [31]. From the nutritional point of view, the n6/n3 ratio is generally used to assess milk fat quality [8]. Diets with a lower n6/n3 ratio are considered healthier for humans [5] due to the reduced risk of many chronic diseases. According to [4], a very high ratio, i.e., excessive amounts of n6 PUFAs, promotes the pathogenesis of many chronic diseases. On the other hand, the increased intake of n3 FAs can lead to a lower risk of cardiovascular diseases [32]. The optimum dietary n6/n3 ratio should be around 1–4:1 [14,33]; however, the ratio in a typical Western diet varies between 10:1 and 20:1 [14]. 

Aside from the n6/n3 ratio, milk fat quality can be evaluated through some proposed indices, such as the atherogenic index, thrombogenic index, health-promoting index, or hypo-/hypercholesterolaemic ratio [34,35,36]. These indices take into consideration the fact that SFAs with chain lengths of 12 to 16 carbons are considered to be atherogenic [37].

According to [34], the atherogenic index (AI) and thrombogenic index (TI) are markers that indicate a potential risk of cardiovascular diseases. They are calculated as follows:

AI = (C12:0 + 4 × C14:0 + C16:0)/(∑MUFA + ∑PUFA);

TI = (C14:0 + C16:0 + C18:0)/[(0.5 × ∑MUFA + 0.5 × ∑PUFA (n6) + 3 × ∑PUFA (n3)) + (n3)/(n6)].

The health-promoting index (HPI) is the inverse of the atherogenic index [9,35]:

HPI = (∑MUFA + ∑PUFA)/(C12:0 + 4 × C14:0 + C16:0);

Hypocholesterolaemic/hypercholesterolaemic ratio (h/H) is calculated according to the following formula [36]:

h/H = (C18:1n9 + C18:2n6 + C20:4n6 + C18:3n3 + C20:5n3 + C22:5n3 + C22:6n3)/(C14:0 + C16:0).

From the health point of view, milk fat with low AI and TI values and, on the contrary, with a high HPI index and h/H ratio is desirable, because it is a sign of a lower risk of cardiovascular diseases [38]. In addition to these indices, ratios of PUFA/SFA and S/P ratio (see the formula below) are used to evaluate the nutritional value of milk fat as well [38].

S/P = (C14:0 + C16:0 + C18:0)/(∑MUFA + ∑PUFA)

On the other hand, a higher proportion of PUFAs in milk fat is connected with the impaired technological properties of milk fat. Increased PUFAs in milk fat can have both positive and negative effects on these properties. Although a positive effect is represented by an improved spreadability of milk fat, a negative effect is represented by an increased susceptibility to oxidation. Thus, to evaluate the effect of a modified proportion of milk FAs on the technological properties of milk fat, the spreadability index (SI) can provide a deeper insight into the quality of milk fat and can be calculated according to the following formula [39]:

SI = C18:1c9/C16:0

Furthermore, an important parameter of milk fat synthesis is the ability of the mammary gland to desaturate FAs originating from the blood (derived from the diet or microbial activity in the rumen) or from de novo synthesis with the help of the stearoyl-CoA desaturase (SCD) enzyme, which catalyses the introduction of a cis-double bond between carbon atoms 9 and 10 of FAs with a chain length of 10 to 18 carbons [3]. To characterize this process, desaturation indices (DI) are calculated from the amount of specific SFAs and corresponding MUFAs as a percentage of the product from the sum of the product and the substrate [40]. The following product and substrate pairs can be taken into calculations:

DI (C14) = (C14:1c9) * 100/(C14:0 + C14:1c9)

DI (C16) = (C16:1c9) * 100/(C16:0 + C16:1c9)

DI (C18) = (C18:1c9) * 100/(C18:0 + C18:1c9)

DI (RA) = (C18:2c9t11) * 100/(C18:1t11 + C18:2c9t11)

## 3. Alternative and Unconventional Feeds Used in Dairy Diets

### 3.1. Macroalgae and Microalgae

Algae belong predominantly to the group of photosynthetic organisms that can grow in a range of aquatic habitats. However, many photosynthetic species (e.g., *Chlorella*, *Scenedesmus*, *Hamaetococcus*, *Spirulina*, and *Nostoc*) are able to grow heterotrophically, using organic substrates as sole sources of carbon [41,42]. Marine algae generally contain a wide spectrum of valuable, biologically active compounds, such as polysaccharides, proteins, PUFAs, various pigments, antioxidants, etc. Considering this spectrum, it predestines them to diverse commercial applications. According to their size, algae are divided into macroalgae (large-sized algae) and microalgae (microscopic single cells) [41].

#### 3.1.1. Macroalgae

Macroalgae (also called seaweeds) generally reside in the littoral zone [43]. According to the presence of specific pigments, macroalgae are divided into three major groups: *Rhodophyta* (red algae), *Phaeophyta* (brown algae), and *Chlorophyta* (green algae) [23,43]. *Ascophyllum nodosum*, *Laminaria* sp., *Lithothamnion* sp., *Macrocystis pyrifera* (giant kelp), *Sargassum* sp., *Palmaria palmata*, and *Ulva* sp. are the main genera and species which have the potential to be used as animal feeds [43]. Although the nutritional value of seaweeds has been reviewed recently [44], it is worth mentioning some specific polysaccharides, such as fucoidan, a sulphated polysaccharide found in the cell walls of brown macroalgae that exerts antitumor, antithrombotic, and antiviral properties [23,45]; laminarin, a polysaccharide isolated from brown seaweed [46]; and ulvan, found in green algae of *Ulva* sp. [47], with various biological activities. Furthermore, macroalgae are also an excellent source of minerals and trace elements, vitamins, and other bioactive compounds such as pigments [23]. Despite having a low content of lipids (1–5%), macroalgae are rich in PUFAs (reviewed in [23,44]), namely, EPAs and AAs, found mainly in brown and red seaweeds [44,48]. However, it should be noted that the chemical and nutritional composition of seaweeds is highly variable, depending on many factors [23,44,48].

Among their diverse applications, seaweeds are also considered to be a suitable feed additive for livestock animals. However, scientific data concerning the effect of macroalgae supplemented into ruminant diets on milk yield and composition are limited, [49,50] reported an increase in milk fat yields in dairy cows supplemented with the calcareous red alga *Lithothamnion calcareum*, which was used as a buffer. Caroprese et al. [51] demonstrated the positive effect of brown alga *Ascophyllum nodosum* on the performance of lactating ewes. They reported increased milk yield and total n3 FAs; the highest n3/n6 ratio was found in milk from ewes supplemented with flaxseed in combination with *Ascophyllum nodosum* compared to controls; furthermore, flaxseed and flaxseed + brown algae decreased AI and TI. Quigley et al. [52] reported an increase in δ-tocopherol in the milk of cows supplemented with *A. nodosum*. On the other hand, the addition of the brown alga *Undaria pinnatifida* (in a mixture of brown seaweed, pinecone oil, and garlic extracts, respectively) or the red seaweed *Gracilaria birdiae* to lactating diets did not affect cows’ or goats’ milk performance, respectively [53,54]. Recently, a positive effect of some algal species on the mitigation of methane emissions in ruminants has been demonstrated. Among macroalgae, the red seaweed *Asparagopsis armata* seems to be particularly efficient in the reduction of methane production [55,56].

#### 3.1.2. Microalgae

Microalgae are a morphologically diverse group of aquatic unicellular or multicellular microorganisms with a range of 0–200 µm. [57]. Generally, microalgae are considered photoautotrophic organisms, whereas a number of species (e.g., *Schizochytrium*) are heterotrophic. Microalgae show high metabolic flexibility and most of them are able to grow under both autotrophic and heterotrophic conditions (mixotrophic algae) [58].

Microalgae produce a wide range of bioactive compounds, such as PUFAs, pigments, and antioxidants, so they can be used as supplements not only in human diets but also in animal diets [59,60]. Indeed, about 30% of the current world algal production is sold for animal feeding purposes [61] and *Spirulina* (*Arthrospira*) and *Chlorella* sp. belong to major microalgae, with possible applications in human and animal nutrition [59]. However, the higher cost of *Chlorella* biomass production limits its wider usage as a protein supplement in animal diets [62]. On the other hand, the content of desirable substances (e.g., PUFAs) in microalgae can be modified by external factors such as temperature or light intensity during cultivation [59,63]. Furthermore, the nutrient profile can be altered by engineering a number of enzymes (desturases and elongases) and transcription factors [64] or by filtering light [65]. Furthermore, the selection of microalgal species plays a role because some strains from the *Nannochloropsis*, *Phaeodactylum*, *Schizochytrium*, *Arthrospira*, and *Thraustochytrium* genera can accumulate high contents of EPA, DHA, and/or γ-linolenic acid [59,63,66,67,68].

*Chlorella* is among the most cultivated eukaryotic microalgae and is used as a food supplement and feed additive. *Chlorella* contains 50–60% crude protein (% dry matter) [69,70] and well-balanced essential amino acids [70]; thus, *Chlorella* represents an excellent novel protein source. Furthermore, an important substance in *Chlorella* seems to be β-1,3-glucan [71]. In addition, *Chlorella* species, e.g., *Chlorella kessleri*, are also notably high in C18:3 (n3) [8]. Spirulina, e.g., *Arthrospira maxima*, or blue-green algae (cyanobacteria), is also known to have a high protein content—60–71% depending on the strain; dried spirulina biomass contains all the essential amino acids, with excellent bioavailability [60,69,72]. Moreover, spirulina is a rich source of carotenoids and FAs and contains about 1.7% of total PUFAs, of which linoleic and γ-linolenic acids account for 45% [23,73,74].

Another microalga that is commercially used as a food supplement or can be used as a supplementary feed additive is the green alga *Haematococcus pluvialis*, which accumulates the carotenoid pigment astaxanthin, and which has been recognized as the richest natural source of this pigment [59]. Astaxanthin is known especially for its strong antioxidant activity that provides various health benefits [75]. *Dunaliella salina* is a halotolerant microalga with potential for use in food and feed applications due to its ability to accumulate large amounts of β-carotene [59,66]; moreover, it can also produce high-quality protein [76]. A green microalga, *Scenedesmus almeriensis*, is a rich source of the carotenoid lutein [66].

Microalgae can positively affect the quality of bovine milk because EPA and DHA can be transferred from the diet into milk [58], so it can be an effective way to reduce the amount of potentially atherogenic FAs in milk, such as lauric, myristic, and palmitic FAs [77]. 

For the manipulation of the FA composition of milk, microalgae can be supplemented into the cow diet in different forms, as microalgal biomass (defatted or full-fat) or oil [69]. There is a relatively large amount of PUFAs in the feed rations of ruminants; however, the transfer of EPA and DHA from the diet into milk can lead to limited results due to the extensive biohydrogenation of these FAs in the rumen (if they are unprotected), which is the reason the transfer efficiency is low [13,58,78,79,80]. Thus, the efficiency of the transfer of unsaturated FAs from feed to milk depends on the effectiveness of their protection in the rumen [81,82]. When some protected sources of n-3 LC PUFAs are involved in the diets of dairy cows, such as protected fish oil, the transfer efficiency of both EPA and DHA into the milk can be increased, although even when feeding animals with food from protected sources, the response to increased EPA and DHA can be still low [13,83]. Biologically active compounds in microalgae are protected thanks to the special composition of the cell wall and cell membranes [82]. Therefore, the addition of marine-derived supplements seems to be an efficient method of producing n3 PUFA-enriched milk. The results of some studies (e.g., [8,84,85]) have shown that the inclusion of microalgae in the diet of dairy cows can affected the FA profile of their milk, mainly in the content of rumenic acid and n3 FAs, the n6/n3 ratio, and the transfer efficiency of DHA from the diet into milk fat. However, in [86] it was observed that changes in milk fat composition depend on the dose of algal supplementations, e.g., graduated doses of microalgae that are rich in DHA in the diets of dairy cows resulted in reduced SFA content, whereas the proportion of PUFAs, C18:2cis9t11, and other FAs were significantly increased [84]. In [8], the n3 FA levels were higher in microalgae-supplemented cows compared to controls, and [79] reported increased levels of DHA in the milk of cows fed a silage-based diet with microalgae supplementation. Similarly, the positive effect of microalgae supplementation on DHA content, DPA, and EPA in the milk of small ruminants has been reported [8,87,88,89].

### 3.2. By-Products of the Food Industry

#### 3.2.1. Okara Meal

In Japan and other parts of eastern and Southeast Asia, soybeans are commonly used to make various foods, such as tofu, soymilk, tempeh, soybean oil, soy flour, and soy sauce. During the processing of soy milk and tofu, the soybeans are ground and water is used to obtain water-extractable fractions. The main residue from this processing is okara [90,91]. About 1.1 kg of fresh soybean curd residue is produced from each kilogram of soybeans processed into soy milk or tofu [91]. The stability of fresh okara is relatively low and should therefore be used quickly. Fresh okara has a high moisture content (from 70% to 80%), which makes it difficult to handle, and it is too expensive to dry it by means of conventional methods [92]. Suitable ways of incorporating okara into feed are currently being sought that would add economic value to the product and eliminate a possible source of contamination [93,94]. Although okara is a highly nutritionally valuable product, it is often landfilled or disposed of via incineration [90], which is accompanied by the release of carbon dioxide [91]. Due to its susceptibility to rot and excessive residue formation, the disposal of soybean curd residues poses a potential environmental problem [95,96,97]. However, soy curd residue is widely recognized for its high nutritional value and excellent functional properties as a relatively inexpensive source of protein [91,98]. There is only a small amount of okara, which is sometimes used for feeding purposes [90]. For example, in the United States, part of the okara, which comes from the production of organically certified soy milk and tofu, is used for feeding purposes in organic herds [99]. Due to differences in sources and processing methods, the nutritional value of okara is very variable. Crude protein concentrations range from 16% to 33%, ether extract from 0.8% to 22%, carbohydrates from 2.6% to 54% and fiber from 4.5% to 58% [91]. In addition, okara is rich in long-chain FAs and the isoflavones daidzein and genistein [100,101]. It was reported that 50% of the total FAs present in freeze-dried and ground okara consisted of C18:2c9c12, which is slightly less than that reported for soybean meal (52% of the total FAs; [101,102]). Several studies have been conducted to investigate the effect of moist okara in diets on ruminants, and no adverse effects on dry matter intake and production performance have been reported [103,104,105,106]. Improved dry matter intake and apparent total tract digestibility of dry matter, organic matter, and NDF have been observed in mature lambs fed with TMR containing wet okara silage [107]. However, all the above-mentioned studies used wet or ensiled okara. Research focused on the use of dried okara (i.e., okara meal) as a protein source in lactating dairy cows is lacking. Recent results have proved that okara meal is a suitable replacement for soybean meal in dairy diets, without negative effects on lactation performance and dry matter intake [99].

#### 3.2.2. Camelina sativa Seeds or Expellers

Camelina (*Camelina sativa* L.) is an oil plant belonging to the *Brassicaceae* family and has often been referred to as “false flax” [108]. This annual or hibernating herb originates from the Mediterranean to Central Asia and thus represents an alternative source in the context of adaptive and resilient ruminant farming systems [109,110]. Camelina is a low-input oil seed crop with very high nutrient efficiency that can grow with limited nitrogen fertilization [111,112]. In addition, camelina has a relatively short growing season of about 4 months and can therefore be integrated into double-growing systems during cold growing periods [113]. Many studies of camelina have highlighted its potential as a promising oilseed crop for sustainable farming systems [111]. A by-product of the oil industry, camelina-rich press-cake is a valuable feed for livestock. This oilseed meal still contains 10% oil, 13% fiber, 5% minerals, and 45% protein [110]. Camelina seed contains 40–44% crude protein and 39–47% fat [114] and is interesting as a source of protein and energy in the ratio of high-yielding dairy cows [112]. It has a similar FA profile to flaxseed and is rich in linolenic acid. Camelina oil contains 20% to 40% C18:3 (mainly α-linolenic acid), 10% to 20% C18:2 (mainly linoleic acid), 12% to 25% C18:1, 13% to 21% C20:1 and 2% to 5% C22:1 [110,114,115,116]. Camelina seeds and their by-products contain antinutritional factors that limit their use in animal feeds. Camelina seeds contain glucosinolates, which induce a lower activity of the thyroid gland and cause disturbances in metabolism. The growing environment of camelina has been reported to influence its glucosinolate content. The main glucosinolates identified in CE and WCS were glucoarabin, glucocamelein, and gluconesliapaniculatin, with slightly lower concentrations in CE [111,117]. Camelina seeds contain sinapine, which is important in plants for the biosynthesis of lignin and flavonoids. However, sinapine has several properties that are undesirable as a constituent in animal feeds [118]. Camelina oil also contains small amounts of C22:1c13 (erucic acid; 2–6% of total FA) [115]. Potential health concerns are associated with the dietary consumption of this FA because experimental studies have demonstrated an association between dietary cis-13 22:1 and heart disease [117]. It is therefore of interest to quantify the magnitude of transfer of this FA from dietary CE or WCS to milk fat in lactating ruminants [117]. Camelina seeds are also relatively abundant in essential amino acids [114], highlighting the potential of camelina as a high-quality feed for ruminants. Several studies have shown that the usage of camelina seeds, cakes, or oil can alter the milk fat composition in cows fed with diets based on maize silage [99], red clover silage [119], or grass silage [116,120]. Similar effects were also observed in goats and sheep [121,122,123].

### 3.3. Tanniferous Plants

Some dietary phenolic compounds can be beneficial for human health. Recognized benefits include the inhibition of oxidative stress and cell destruction associated with degenerative diseases and modern lifestyle [124], protection against oxidative damage [125], and the prevention of chronic diseases [126].

Important amounts of phenolic compounds, especially flavonoids, are contained in grape seeds [127]. Grape oil flour has been studied for its reducing effect on methane emissions. It has been found that the addition of grape seed meal favorably modulates rumen fermentation by reducing methane production, without adversely affecting fiber degradation. It can help to stabilize rumen pH in a grain-rich diet by forming complexes with some rapidly degradable carbohydrates [128]. The whole grain seeds contained in the lamb diet cause higher weight gain. Increasing the levels of grape seed meal were shown to reduce saturated fatty acids, increase unsaturated fatty acids, and improve the eating properties of meat, with good atherogenicity and thrombogenicity indices [129]. A number of experiments with ewes have examined the effects of the dietary classification of grape seeds on milk production and milk quality [130]. Experiments have suggested that grape seeds may be useful for increasing the concentration of polyunsaturated fatty acids, with potential health benefits for consumers [131]. The addition of grape seeds reduced the extent of oxidation of total unsaturated fatty acids in milk [132].

Phenols, especially tannins, can inhibit or counteract the rumen biohydrogenation of polyunsaturated FAs (PUFAs), such as ALA and LA [133,134]. Depending on the effects on rumen biohydrogenation, tannins are commonly divided into two main classes. These are insoluble condensed tannins that have a very high molecular weight. Their formation occurs through the polymerization of flavon-3-ol and is characterized by an inhibitory effect on rumen biohydrogenation [135]. On the other hand, soluble hydrolyzable tannins are non-flavonoid compounds that also appear to have a modulatory effect on rumen biohydrogenation [135]. Tannins inhibit microbes that are involved in rumen biohydrogenation through mechanisms that are not yet clear. The fact remains that the effects of tannins are modulated by the potential adaptation of the rumen microflora to these phytochemicals, as well as by chemical composition, macronutrient interactions, as well as the dose [135,136]. Based on the results of in vitro and in vivo studies, it was observed that tannins exert differing effects on the rumen bacteria involved in lipolysis, the first step of rumen biohydrogenation, and specifically the conversion of ALA and LA to vaccenic acid (VA) and VA to C18:0 [135]. Various theories apply to this issue. One is that tannins can exhibit toxic effects by altering the permeability of bacterial membranes [137]. Another theory is that tannins reduce rumen biohydrogenation by inhibiting rumen microbial activity, rather than by inhibiting their enzymatic activity. This inhibition is not constant but affects different rumen biohydrogenation steps at different rates [138]. Selected additives may also improve the feeding efficiency of dairy cattle. The potential of tannins (hydrolyzable and condensed) to increase the utilization of proteins in the diet of ruminants is associated with their ability to bind proteins in the rumen and thus prevent their excessive microbial degradation. Tannin–protein complexes are dissociated in acidic pH in the abomasum or under alkaline conditions of the small intestine, releasing the protein for digestion and absorption [139]. Some tannins added to ruminant diets have also been shown to reduce methane production in the rumen without adversely affecting the efficiency of fermentation in the rumen [140,141,142].

### 3.4. Herbs and Spices

#### 3.4.1. Oregano

Herbs or medicinal plants, including oregano (*Origanum vulgare*), contain essential oils (EOs), volatile plant secondary metabolites that give plants a characteristic odor and taste [143]. They protect plants from damage by microorganisms, herbivores, and UV-B radiation [144]. Essential oils have strong and non-specific antimicrobial properties; however, microbes show different levels of sensitivity to EOs [143,145]. Oregano is a perennial herb that grows in Eurasia and North Africa. The EO content of Greek oregano (*Origanum vulgare* ssp. *hirtum*) is high, approximately 4% of the dry matter [146,147]. Another subspecies of oregano, wild marjoram (*Origanum vulgare* ssp. *vulgare*), has naturally lower levels of EOs, with a concentration of only 0.2% dry matter and up to 1% dry matter [146,147]. The effects of low-EO oregano (*O. vulgare* ssp. *vulgare*) and high-EO oregano plant material (*O. vulgare* ssp. *hirtum*) on methane production, rumen fermentation, nutrient digestibility, and milk fatty acid composition were studied recently [148]. Dry matter intake was not affected by feeding with low- or high-EO oregano, suggesting that doses of oregano administered to cows did not affect digestibility. DMI was not affected by the administration of oregano [149] or oregano EO [150]. As in recent studies [148,149,150,151,152], it does not affect milk yield. The effect on milk composition has only been studied in some studies that report either increased or decreased fat and protein contents [149,150,152]. Oregano with or without a high level of EO does not appear to affect feed conversion efficiency, despite the fact that the literature refers to carvacrol and thymol as being similar to ionophore antibiotics [153]. In contrast, improved feeding efficiency with 3.5% FCM per kilogram of DMI [149] and a kilogram of milk per kilogram of DMI has been previously reported [151].

#### 3.4.2. Hop Plants

Hops (*Humulus lupulus* L.), which are added to beer as preservatives, are a potential additive to reduce rumen CH_4_ emissions and protein degradation. The antimicrobial components of hops—including humulone, lupulon (commonly called α- and β-acid), and their isomers—inhibit lactic acid bacteria, which spoil beer [139]. The types of hyper-NH_3_-producing bacteria present in the rumen are sensitive to the antimicrobial components of hops. Hops used in in vitro experiments reduced the degradability of dry matter and crude protein in the rumen without affecting the digestibility of DM and CP [154]. The inclusion of hops at between 400 and 800 mg/L of culture fluid in in vitro rumen incubations reduced CH_4_ production and the acetate/propionate ratio [155,156]. Hops thus appear to be another promising natural feed additive in reducing the production of CH_4_ in the rumen [139]. When used in combination, hop pellets and oak extracts can complement each other to reduce rumen CH_4_ production and NH_3_ efflux in ruminants [156]. There is a lack of published information on the effect of hops on milk production and milk composition in cows. Hop pellets did not affect milk production or milk composition. In contrast, dietary supplementation with a combination of hop pellets and tannin extracts increased milk production and protein yield. Similarly, no published scientific work is available on the effect of hops on rumen biohydrogenation. Hop α- and β-acids inhibit most Gram-positive bacteria in a similar manner to monensin [155]. The antimicrobial activity of hops is similar to that of monensin. Therefore, the inhibition of FA biohydrogenation, leading to higher levels of UFA in milk, could be expected. Hops alone at the tested dose had no significant effect on the fatty acid profile of milk. This dose was either too low or the rumen microbes were adapted to hops [139].

### 3.5. Other Plants

#### 3.5.1. Cactus Cladodes

Cacti of the genus *Opuntia* (commonly referred to as prickly pear cactus) are an important source of feed for ruminants in the semi-arid regions of Brazil and other arid regions around the world. The ability of cacti to thrive in severe drought conditions is remarkable [157]. Cactus has a high moisture content in its cladodes and can therefore meet a significant part of the water requirements for livestock [158]. In contrast to traditional feeds, cladodes from *Opuntia* spp. contain approximately 250 g of neutral detergent fiber (NDF) and 500–600 g of non-fiber carbohydrates (NFC) per kg of dry matter (DM) [159]. Cactus cladodes thus contain high levels of digestible energy, which leads to a reduction in the proportion of concentrates, such as cereal grains, in ruminant diets. Several studies have addressed the effects of cactus feeding on rumen fermentation and production performance [159]; however, information on the effect of cactus cladodes on the FA composition of bovine milk is scarce. In cows fed with cactus cladodes, a low proportion of 18:0 in milk fat (<5 g/100 g total FAs) was observed, regardless of the composition of the basal diet [160]. This observation could be associated with a low lipid content in cladodes (10–15 g/kg DM), which in turn would lead to low rumen production of 18:0 due to a reduced dietary intake of PUFAs for rumen biohydrogenation [160]. However, the 18:0 content of the milk fat of cows fed with a sugar cane diet was only slightly lower than the levels normally reported for a conventional dairy diet [161,162]. This fact suggests that other mechanisms may account for the reduced 18:0 content observed in the milk fat of cows consuming cacti. A possible mechanism could be faster permeability of the rumen digestive tract, which is expected to shorten the exposure time of feed particles to rumen bacteria, leading to less complete BH of dietary PUFAs [163]. This hypothesis is supported by the presence of large feed particles in the feces of cows fed with a diet containing high proportions (350–500 g/kg DM) of cactus cladodes [148]. Alternatively, the presence of numerous phenolic compounds in the cladodes of *Opuntia* spp. [164,165], including *Opuntia stricta* [166,167], can alter the microbial population of the rumen by inhibiting the bacterial species responsible for the last step in rumen biohydrogenation [135]. This finding, regardless of the mechanisms underlying the 18:0 reduction in the milk fat of cows fed with cactus cladodes, suggests that combining cladodes with PUFA-rich vegetable oils may increase the rumen accumulation and outflow of the 18:1 trans-11 for the mammary synthesis of cis-9, trans-11-conjugated linoleic acid (CLA), thereby improving the nutritional quality of milk fat [81].

#### 3.5.2. Blue Lupine

Several species of lupine (e.g., *Lupinus albus*, *Lupinus luteus*, and *Lupinus angustifolius*) have been commonly used in animal diets. To date, the dietary use of lupins in ruminants has been studied mainly for its high protein content as a potential substitution for soybean meal [168]. Indeed, lupines contain comparable amounts of protein with a similar amino acid profile, and the digestibility of lupine protein is comparable to the digestibility of soy protein [169]. On the other hand, lupines have a higher fiber content, which is nutritionally favorable compared to soybeans [170,171]. Furthermore, studies on lupine as a source of valuable nutrients for ruminants are of growing interest due to their positive effect on the animal production and biological value of animal products, e.g., on the FA profile of milk fat, as documented for *L. albus* [171,172,173]. In recent years, blue lupine (*L. angustifolius*) has been widely introduced as a potential source of livestock feed. Its seeds are high in protein, at 330 g/kg dry matter, and in non-starch polysaccharides, at 400 g/kg dry matter, composed mainly of a non-cellulosic polymer and pectin polysaccharides [168,171].

#### 3.5.3. Olive Leaves and by-Products

The olive tree (*Olea europaea* L.), a member of the *Oleaceae*, is a slow-growing evergreen tree that is essentially native to the Mediterranean climate [174]. The term olive leaves refers to a mix of leaves and branches obtained from olive tree pruning and olive harvesting and cleaning [175]. Such a mix appears to be a copious by-product, representing (approximately) 10% of the total weight of harvested olives [176], and accounting for almost 5% of overall yield from olive oil by-products [177]. These residues could be useful for the feeding of small ruminants when there is a lack of availability of forages [178]. Actually, olive leaf by-products may play an important role in the integrated use of the available resources and reducing environmental impacts [178]. The increasing amount of these residues represents a major problem due to their adverse effects on environmental sustainability [179]. This underutilized biomass could be regarded as a valuable resource in the food sector, as it is useful in the production of additives, credited with prominent antioxidant, anti-inflammatory, and antimicrobial activities [179,180]. The high biological value of this by-product is due to the presence of well-known and well-characterized phenolic bioactive compounds, such as caffeic acid, tyrosol, hydroxytyrosol, flavones (apigenin, kaempferol, and luteolin), and oleuropeosides (oleuropein and verbascoside) [181]. Olive leaves can be used as a resource in the zootechnical field as a dietary supplement for farm animals. Recent studies show that olive leaves can induce positive effects when integrated into animal diets [174]. Several factors (e.g., sampling period, cultivar, age of the olive tree, climatic changes, and the process of obtention) affect olive leaf composition. The average composition of an olive leaf is 49.8% moisture, 7.6% proteins, 1.1% lipids, 4.5% minerals, and 37.1% carbohydrates. Olive leaves are characterized by a high level of fiber and lignin [182].

## 4. Effect of Alternative and Unconventional Feeds on Selected Fatty Acids and Health Properties of Milk Fat

The effects of alternative and unconventional feedstuffs used in dairy diets on selected FAs, their sums, ratios, and indices describing the health and technological properties of milk fat, are described in Table 1, Table 2, Table 3 and Table 4. The majority of these feeds were included in TMR diets based on preserved feeds, mainly silages/baleages made from maize, grasses, legumes, sorghum, or their mixtures or hay (grass, lucerne) and concentrated feeds, represented predominantly by maize, barley/wheat, and soybean meal (see Table 1). The rate of inclusion of these feeds into the animals’ diets differed according to the major nutrients for which they were included into the diet (sources of protein, minerals, FA, etc.), the feeds’ properties (effect on palatability, presence of antinutritive substances), or their availability and form (extracts, natural feeds). Although macroalgae, such as *Ascophyllum nodosum*, are used mainly for their high content of macro- and microminerals, polysaccharides, polyphenols, and bioactive peptides [183], microalgae are rich in crude protein and/or long-chain PUFAs, especially EPA, DHA, and linoleic and/or γ-linolenic acid [59,63,66,67,68]. The algal components were generally given in amounts ranging from 100 to 310 g/d on an as-fed basis (see the Table 1), except for the situation in which the microalgae were used as a protein substitution for soybean meal in daily amounts ranging from 1.12 to 1.63 kg DM/d [67]. As mentioned, microalga; supplementations are used to increase the content of n3 FAs in milk, mainly ALA, EPA, and DHA, and to improve the n6/n3 ratio [59,63,66,67,68]. It seems that the highest potential for such improvement is in *Schizochytrium* sp. and in a combination of *Chlorella* + *Nannochloropsis* (Table 2 and Table 3).

Compared to algal products, industrial by-products such as okara meal or camelina expellers, as protein sources, were incorporated into dairy diets in higher proportions, at 150 g/kg DM or 2.4 kg/d as-fed, respectively. In the case of the inclusion of whole camelina seeds, the rate was lower, at 42 g/kg DM, due to a presence of antinutritive substances, as described above. Furthermore, these products are also rich in C18:1c9, C18:2n6, and C18:3n3 (see Table 1), so they seem to be especially efficient in increasing the total PUFA and n3 FA contents in milk fat [117,119], resulting in favourable n6/n3 ratios (Table 3).

Tannins are used primarily for the modification of biohydrogenation processes and protein digestion in the rumen; some of them have also been used to reduce methanogenesis. However, tannin extracts are expensive and may reduce the palatability of diets. This is why various tanniferous plants were tested as an alternative to tannin extracts [183]. Aside from their phenol contents, tanniferous plants are rich in C16:0, ranging from 19.7 to 47 g/100 g FA, according to plant species, and in C18:1c9, C18:2n6, and C18:3n3 (see Table 1). They can also induce changes in milk FAs by altering the course of rumen biohydrogenation. These effects were mainly obvious in minor FAs (C15:0–C17:0), the long-chain FAs (mainly ALA), and intermediates of ruminal biohydrogenation (VA, RA) [183]. Among the reviewed tanniferous compounds, oak tannins (heartwood) seem to be the most efficient in reducing SFAs and increasing MUFA and PUFA contents (see Table 3). Herbs and spices such as oregano or garlic contain EOs that are thought to be efficient in lowering methane emissions, which is the primary reason why they have been tested in dairy diets. Furthermore, the production of CH_4_ represents losses of dietary energy ranging between 2% to 12%, depending on the diet [184]. Due to their strong antimicrobial properties, EO can also alter rumen fermentation by inhibiting fibrolytic bacteria or by reducing the abundance of Archaea [148], thus improving the digestibility of nutrients and increasing the amount of energy available for animal production. After supplementation with high-EO oregano, no effect on milk FA was observed [148]. On the other hand, feeding with low-EO oregano increased the content of ALA in milk fat [148]. The discrepancies in animal responses to EOs suggests that not only the dose, but also the form in which the EOs are supplied (EO as a supplement vs. as a natural plant/forage component), is important [148,151,152]. Therefore, the form of inclusion of herbs and spices into dairy diets should be also considered when examining their effects on the specific or secondary aspects of animal production, such as the FA profile of milk. Compared to oregano EOs, the information about the effect of hops on rumen fermentation and milk performance is scarce. Although the inhibitory effects of hops’ bitter-tasting acids on most Gram-positive bacteria have been reported [155] no changes in milk FA profiles, nor in CH4 emissions, DM, or organic matter, were noted, suggesting that the dose tested (56 g/kg DM, Table 1) was too low [139]. Further studies are needed to clarify the effect of hops or their active substances on the course of fermentation, biohydrogenation, and methanogenesis in the rumen.

Among the other feedstuffs, cactus cladode silage was included in the diet in the amount of 340 g/kg DM and, aside from its main nutrients, it was rich in C16:0 and linoleic acid and ALA. Feeding with cactus cladode silage seems to be beneficial in terms of a decreased content of milk SFAs and increased contents of MUFAs and PUFAs, namely, VA and RA. Although the n6/n3 ratio was relatively high, the AI was low due to the low contents of atherogenic FAs C12:0, C14, and C16:0 (Table 1, Table 2, Table 3 and Table 4).

Generally, among the reviewed feeds, some microalgae of *Schizochytrium* spp. [85,86], *Chlorella vulgaris* [77], rumen-protected algae [79,185], and blue lupine can be thought to be efficient in improving the n6 FA content in milk fat (Table 3). On the other hand, feeding with tanniferous plants, by-products, *Spirulina platensis*, *Ascophyllum nodosum*, *Aurantiochytrrium limacinum* [186], and *Schizochytrium* spp. [187] resulted in low n6 FA contents in milk fat (Table 3). In the case of n3 FAs, low contents in milk were found after using *Ascophyllum nodosum*, *Aurantiochytrium limacinum* [186], *Spirulina platensis*, and camelina expeller [117] for the feeding of dairy cows. On the other hand, tanniferous plants, some *Schizochytrium* spp. [86,187], and camelina expeller [119] feeding resulted in elevated n3 FA in milk. The corresponding n6/n3 ratio varied from values as low as 0.39 or 0.45, found in whole camelina seeds and camelina expeller, respectively, to values as high as 11.2 in blue lupine or 6.78 in *Schizochytrium limacinum* in [77].

Generally, among the reviewed feeds, the highest content of C12:0 was found in milk fat after feeding with all tanniferous plants, fed in the form of plant components (not extract) [183], and the lowest content was found after feeding with cactus cladode silage. On the other hand, no clear effect of algal supplements on C12:0 was observed (Table 2). Similar findings were also noted for C14:0 and C16:0 FAs, resulting in a high content of SFAs in milk after feeding with tanniferous plants (except for tannin extract and black current), ranging from 70.1 to 71.8 g/100g FA. However, the highest content of SFAs in milk fat was observed after feeding with dry oregano plants, regardless of the content of EOs, due to the high content of C16:0 (Table 2 and Table 3). The lowest content of SFAs was found when feeding with cactus cladode silage (53.4 g/100 g FA), followed by rumen-protected algae (55.63 g/100 g FA) [185] and camelina expeller (56.05 g/kg FA) [117] (Table 3). The highest content of MUFAs was found when feeding with camelina expeller (37.92 g/100 g FA) [117] and cactus cladode silage (36.40 g/100 g FA) [160]. A low content of MUFAs was observed after feeding with the combination of oregano plants, macroalgae *Acophyllum nodosum* [188], *Spirulina platensis* [8], and *Schizochytrium* spp., as reported in [86] (Table 3). Feeding all four of these feeds resulted in a lower content of oleic acid (C18:1c9), suggesting lower desaturation from C18:0 [189] (see Table 2). The highest content of PUFAs in milk fat from all reviewed feeds was found in *Schizochytrium* spp., at 9.82 g/100 g FA, mainly due to high contents of DHA, ALA, and LA [86]. Furthermore, camelina expellers and cactus cladode silage also seem to be efficient in increasing the PUFA content in milk (Table 2 and Table 3). Moreover, rumen-protected microalgae [79], okara meal, oak tannin extract and hops have the potential to improve the PUFA content in milk. On the other hand, the response in milk PUFA levels to feeding with various algal feeds was inconsistent (compare values in Table 3), suggesting that the transfer of desirable FAs from feed into milk can be influenced by many factors, such as the composition of the basal diet and the interaction of supplements with the forage type, the inclusion level, the length of supplementation, and the response of the rumen microflora to the dietary factors applied [190]. Furthermore, in a recent study, Mavrommatis et al. [190] suggested that the transfer efficiency of DHA and DPA can be influenced by the phase of lactation. A low PUFA content in milk was found after feeding with dry oregano forage regardless of the EO content, blue lupine, some tanniferous plants (hazel and silver birch), macroalgae [188], and *Spirulina platensis* [8,67] (see Table 3).

For the evaluation of health and technological properties of milk fat, various indices can be used (see Table 4). However, in some indices there are limitations that should be mentioned. In the case of AI, and inversely HPI, the calculation includes only selected SFAs (C12:0, C14:0 and C16:0) which were thought to be hypercholesterolaemic [31], and the sum of n3 and n6 PUFAs and MUFAs. Some dietary interventions, such as supplementing the diet with feeds rich in long-chain n3 PUFAs, inhibit the saturation of PUFAs and various isomers of C18:1 to C18:0 in the rumen, resulting in a subsequent decrease in C18:1c9, the main MUFA, in milk, which is produced during the desaturation process in the mammary gland from the above-mentioned substrate FA (C18:0) [189], and thus provides a misleading AI value. Because the usage of AI persists in recent publications, e.g., [168,189], it is necessary to interpret and evaluate AI values with regards to biohydrogenation processes in the rumen.

In the case of the h/H ratio, the main problem is that the majority of scientific studies presenting the FA profile in milk after dietary interventions are lacking in one or more of the FAs needed for the calculation of the index. Indeed, of the feeds evaluated in the present review, only two sources contained all the data necessary for the calculation of the h/H ratio. Furthermore, compared to earlier findings [34], only two FAs, C14:0 and C16:0, have been recently thought to be hypercholesterolaemic [36].

Although the DI can be calculated from various pairs of product–FA substrates, the DI (C14) is suggested to be the best expression of SCD enzyme activity because C14:0 is almost exclusively produced during de novo synthesis in the mammary gland and C14:1c9 is almost exclusively produced during the desaturation process [191,192].

Of the feedstuffs described above, the lowest AI (1.39, Table 4) of all was observed in cactus cladodes, due to their low contents of atherogenic FAs C12:0, C14, and C16:0. Among the algal feeds, a low AI (1.90) was found in rumen-protected algae [185], suggesting that rumen-protected forms of algal supplementation can be suitable not only from the view of the modification of milk FAs, but also from the dietetic point of view, because they eliminate the negative effects of algal products on feed intake, rumen fermentation, and deteriorations in milk yield and quality [58,183]. On the other hand, the highest AI was observed in *Spirulina platensis* (6,8) [77], the *macroalga Ascophyllum* (4.12) [187], and *Schizochytrium* spp. (3.28), [86], suggesting that not all algal supplements can have a positive effect on the health parameters of milk fat. However, recent findings suggest that feeding with algal products rich in DHA can decrease the relative abundance of some bacteria in the rumen (*R. flavefaciens*, *B. proteoclasticus*, *F. succinogenes*, and *S. bovis*), whereas the relative abundance of other bacteria remained unaffected (*R. albus* and *B. fibrisolvens*) [190]. Furthemore, dietary DHA inhibits the final step in the rumen biohydrogenation process, which results in the accumulation of trans-C18:1 FAs, such as C18:1t11 and C18:1t10 [190]. The accumulated trans-C18:1 FA can inhibit specific bacteria that convert C18:1 into C18:0 [190]; thus, the content of C18:1t11 in milk can be increased, as well as the content of C18:2c9t11, which is converted from the C18:1t11 during the desaturation process in the mammary gland [189]. Elevated contents of C18:2c9t11 were also observed in our review in algal feeds that were rich in DHA.

**Table 1:** Effect of alternative and unconventional feedstuffs used in dairy diets on selected milk fatty acids—composition of basal diets, rate of supplementation, and concentrations of selected nutrients.

Feed Supplement	Basal Diets	Inclusion Rate	Concentration of Main Nutrients of Interest	Source
**Macroalgae**				
*Ascophyllum nodosum*	TMR based on a mixed diet—mostly grass and legume baleages, soybean meal, maize, barley, wheat middlings, roasted soybean	170 g/d (as-fed)	NDF–539 g/kg DM Iodine–820 mg/kg DM C16:0–2.73 g/kg DM C18:1c9–5.59 g/kg DM C18:2n6–1.48 g/kg DM	[188]
**Microalgae**				
*Aurantiochytrium limacinum*	TMR based on maize silage, ryegrass, and lucerne hay and concentrate containing soy protein, wheat bran, dehulled sunflower meal, maize meal and germs, molasses, cotton seed, barley flakes, sorghum meal	150 g/d (as-fed)	C22:6n3–160 g/kg	[193]
*Aurantiochytrium limacinum*	TMR based on maize silage, soybean meal, sunflower dehulled seed meal, maize gluten meal, flaked soybean, hydrogenated palm oil, maize and barley flakes, maize and sorghum meals, rye grass hay, and dehydrated lucerne hay	100 g/d (as-fed)	C22:6n3–160 g/kg	[186]
*Schizochytrium limacinum*	TMR based on ryegrass and sorghum silages, cheese whey and concentrate containing maize, soybean meal, and dry distillers grains	144 g/d (as-fed)	C22:6n3–140 g/kg	[77]
*Schizochytrium* spp.	TMR based on maize silage, lucerne hay, cotton seed, molasses and concentrate containing maize, steam-flaked maize, soybean meal, cotton meal, soy hulls, DDGS, and maize gluten meal	255 g/d (as-fed)	C22:6n3–176.4 g/kg DM	[85]
*Schizochytrium* spp.	TMR based on grass and maize silage, standard concentrate and soybean meal	43 g/kg DM intake	C22:6n3–198 g/kg DM (378 g/kg FA)	[187]
*Schizochytrium* spp.	diet based on grass hay and concentrate containing cracked corn, soybean meal, and pelleted dehydrated lucerne	310 g/d (as-fed)	C22:6n3–370 g/kg FA	[194]
*Schizochytrium* spp.	diet based on lucerne hay and concentrate containing wheat, cold-pressed canola meal, and dry molasses	250 g/d (as-fed)	C22:6n3–200 g/kg DM	[86]
*Spirulina platensis*	diet based on grass silage and concentrate containing sugar beet pulps and molasses	1.12 kg DM/d	CP–693 g/kg DM C16:0–45.6 g/100 g FA C18:2n6–23.4 g/100 g FA C18:3n6–19.9 g/100 g FA	[67]
*Spirulina platensis*	diet based on lucerne hay, corn silage, and grain mix containing wheat, maize, extracted sunflower, and soybean meal and wheat bran	150 g/d (as-fed)	C16:0–36.2 g/100 g FA C18:2n6–26.2 g/100 g FA C18:3n6–22.7 g/100 g FA	[8]
*Chlorella vulgaris*	diet based on grass silage and concentrate containing sugar beet pulps and molasses	1.35 kg DM/d	CP–586 g/kg DM C16:0–15.8 g/100 g FA C18:2n6–48.5 g/100 g FA	[67]
*Chlorella vulgaris + Nannochloropsis gaditana*	diet based on grass silage and concentrate containing sugar beet pulps and molasses	0.81 and 0.82 kg DM/d, respectively	CP–485 g/kg * C16:0–20 g/100 g FA * C18:2n6–24.9 g/100 g FA * C20:5n3–9.6 g/100 g FA *	[67]
Rumen-protected macroalgae (Algamac-3050; Aquafauna Bio-Marine Inc., Hawthorne, CA, USA)	TMR based on maize and grass silage, hay, barley, maize distillers grain, maize, and soybean meal or pasture	calculated to provide 200 g/d of total lipids and cca. 65 g/d LC-PUFA	C16:0–25.02 g/100 g FA ** C18:0–32.48 g/100 g FA ** C22:6n3 –24.23 g/100 g FA	[185]
Rumen-protected microalgae DHA-rich microalgae protected with the inert fat (not specified)	TMR based on mixed haylage, maize silage, grass hay, barley, maize, soybean meal, and distillers grains	100 g/d (as-fed)	C22:6n3 –22.28 g/100 g FA	[79]
**By-products**				
Okara meal	TMR based on grass baleage, molasses, maize, soyhulls	150 g/kg DM	CP–32.9 g/kg DM EE–10.5 g/kg DM C18:1c9–19.0 g/kg DM C18:2n6–58.0 g/kg DM C18:3n3–10.6 g/kg DM	[99]
Camelina expeller	TMR based on grass and maize silages, grass hay, barley, maize gluten meal, soybean meal, maize DDGS	95 g/kg DM	C18:1c9–131.4 mg/g FA C18:2n6–223.4 mg/g FA C18:3n3–319.8 mg/g FA	[117]
Camelina expeller	diet based on red clover silage and concentrate containing barley, wheat, sugar beet pulp, molasses, cereal bran, and rapeseed and sunflower oil	2.4 kg/d (as-fed)	NA	[119]
Camelina seeds whole	TMR based on grass and maize silages, grass hay, barley, maize gluten meal, soybean meal, maize DDGS	42 g/kg DM	C18:1c9–121.4 mg/g FA C18:2n6–185.9 mg/g FA C18:3n3–367.7 mg/g FA	[117]
**Tanniferous plants**				
Oak tannin extract	TMR based on maize and grass silages, dehydrated lucerne, rapeseed meal, rolled barley, sugar beet pulp, and extruded linseed	169 g/kg DM	NA	[139]
Hazel	for all: mixed diet based on grass and maize silages, soybean meal, sugar beet pulps, and pellets (lucerne + leaves of one of the given plants)	calculated to reach a total extractable phenol content of 60 g/kg DM	C16:0–27 g/100 g FA C18:1c9–16.1 g/100 g FA C18:2n6–21.6 g/100 g FA C18:3n3–19.2 g/100 g FA	[183]
Silver birch			C16:0–47 g/100 g FA C18:1c9–9.1 g/100 g FA	
Black current			C16:0–28.5 g/100 g FA C18:2n6–14.2 g/100 g FA C18:3n3–35.9 g/100 g FA	
Grape wine			C16:0–30.0 g/100 g FA C18:1c9–9.3 g/100 g FA C18:2n6–17.9 g/100 g FA C18:3n3–24.8 g/100 g FA	
Wood avens			C16:0–19.7 g/100 g FA C18:1c9–19.2 g/100 g FA C18:2n6–24.9 g/100 g FA C18:3n3–22.5 g/100 g FA	
Rosebay willow			C16:0–26.8 g/100 g FA C18:1n9–11.6 g/100 g FA C18:2n6–21.3 g/100 g FA C18:3n3–24.0 g/100 g FA	
**Herbs and spices**				
Hops	TMR based on maize and grass silages, dehydrated lucerne, rapeseed meal, rolled barley, sugar beet pulp, and extruded linseed	56 g/kg DM	NA	[139]
Oregano low in EO (0.12%) (*Origanum vulgare* ssp. *vulgare*)	TMR based on clover-grass and maize silages and concentrates	53 g/kg DM	Carvacrol–31.1 % of total EO Thymol–22.7 % of total EO	[148]
Oregano high in EO (4.21%) (*Origanum vulgare* ssp. *hirtum*)	TMR based on clover-grass and maize silages and concentrates	21 g/kg DM	Carvacrol–35 % of total EO Thymol–41 % of total EO	[148]
**Others**				
Cactus cladode silage	TMR based on sorghum silage, maize, and soybean meal and oil	340 g/kg DM	C16:0–27.8 g/100 g FA C18:2n6–31.7 g/100 g FA C18:3n3–16.5 g/100 g FA	[160]
Blue lupin	TMR based on grass and maize silage, beet pulp, brewers grains, wheat, extracted rapeseed, and soybean meal	94 g/kg DM	CP–345 g/kg DM C16:0–11.2 g/100 g FA C18:1c9–26.5 g/100 g FA C18:2n6–31.8 g/100 g FA C18:3n3–6.58 g/100 g FA	[168]

TMR—total mixed ration, NDF—neutral detergent fiber, DDGS—dried distillers grains with solubles, CP—crude protein, DM—dry matter, FA—fatty acid, LC-PUFA—long chain polyunsaturated fatty acid, EE—ether extract, NA—data not available, EO—essential oil, C16:0—palmitic acid, C18:1c9—oleic acid, C18:2n6—linoleic acid, C18:3n3—α-linolenic acid (ALA), C18:3n6—γ-linolenic acid, C20:5n3—eicosapentaenoic acid (EPA), C22:6n3—docosahexaenoic acid (DHA). * calculated from analytical results for *Chlorella vulgaris* and *Nannochloropsis gaditana* according to their ratio in the supplement (1:1). ** the high content of saturated FA probably comes from the coating (non-specified inert fat).

**Table 2:** Effect of alternative and unconventional feedstuffs used in dairy diets on selected milk fatty acids (g/100 g FA).

	Fatty Acids (g/100 g FA)									
Feed Supplement	C12:0	C14:0	C16:0	C18:1c9	C18:2c9t11 (RA)	C18:2n6 (LA)	C18:3n3 (ALA)	C20:5n3 (EPA)	C22:6n3 (DHA)	Source
**Macroalgae**										
*Ascophyllum nodosum*	3.98	12.49	35.19	13.37	0.48	NA	0.47	0.07	NA	[188]
**Microalgae**										
*Aurantiochytrium limacinum*	3.31	11.53	30.11	17.60	0.86	2.42	0.32	0.05	0.37	[193]
*Aurantiochytrium limacinum*	3.92	12.56	34.07	17.92	0.60	1.83	0.25	0.03	0.10	[186]
*Schizochytrium limacinum*	2.29	8.61	23.84	23.65	1.41	3.50	0.36	0.02	0.14	[77]
*Schizochytrium* spp.	3.78	13.66	24.85	14.87	NA	4.21	0.19	0.04	0.53	[85]
*Schizochytrium* spp.	2.07	8.01	27.70	17.60	1.00	1.37	0.42	NA	1.10	[187]
*Schizochytrium* spp.	4.92	13.98	27.58	7.02	1.38	NA	0.53	0.36	1.15	[194]
*Schizochytrium* spp.	3.54	12.80	34.60	NA	1.78	3.65	0.69	0.11	0.60	[86]
*Spirulina platensis*	3.53	11.50	29.70	19.10	0.44	1.92	0.46	0.04	NA	[67]
*Spirulina platensis*	4.37	14.99	37.84	15.60	0.85	1.62	0.26	0.03	0.02	[8]
*Chlorella vulgaris*	3.32	10.70	27.90	19.00	0.39	3.41	0.57	0.07	NA	[67]
*Chlorella vulgaris* *+ Nannochloropsis gaditana*	3.43	11.10	28.70	18.60	0.45	2.41	0.53	0.21	NA	[67]
Rumen-protected macroalgae (Algamac-3050; Aquafauna Bio-Marine Inc., Hawthorne, CA, USA)	2.85	8.93	26.04	18.64	0.87	2.11	0.51	0.07	0.20	[185]
Rumen-protected microalgae DHA-rich microalgae protected with inert fat (not specified)	NA	NA	NA	NA	3.59	2.43	0.35	0.05	0.22	[79]
**By-products**										
Okara meal	3.78	11.70	30.60	14.70	0.53	1.92	0.53	0.04	NA	[99]
Camelina expeller	3.09	11.91	25.22	12.63	1.39	1.72	0.44	0.03	0.01	[117]
Camelina expeller	3.08	11.90	26.80	NA	1.33	NA	1.06	0.10	0.00	[119]
Camelina seeds whole	3.71	12.12	27.86	13.05	0.66	1.95	0.48	0.04	0.00	[117]
**Tanniferous plants**										
Oak tannin extract	3.25	12.05	26.19	23.09	1.22	2.25	1.46	NA	NA	[139]
Hazel	4.59	15.30	38.10	NA	0.51	1.32	0.83	NA	NA	[183]
Silver birch	4.96	15.20	36.80	NA	0.46	1.35	0.75	NA	NA	
Black current	4.05	14.50	34.60	NA	0.56	1.46	1.00	NA	NA	
Grape wine	4.37	15.20	36.10	NA	0.53	1.43	0.89	NA	NA	
Wood avens	4.64	15.40	35.10	NA	0.54	11.48	0.95	NA	NA	
Rosebay willow	4.61	15.40	35.00	NA	0.49	1.52	0.98	NA	NA	
**Herbs and spices**										
Hops	3.24	12.06	26.77	23.02	1.30	2.18	1.33	NA	NA	[139]
Oregano low in EO (0.12%) (*Origanum vulgare* ssp. *vulgare*)	NA	NA	38.72	14.35	0.35	NA	0.72	NA	NA	[148]
Oregano high in EO (4.21%) (*Origanum vulgare* ssp. *hirtum*)	NA	NA	36.87	15.12	0.52	NA	0.83	NA	NA	[148]
**Others**										
Cactus cladode silage	1.96	8.03	21.50	20.20	2.70	2.90	0.41	0.02	NA	[160]
Blue lupin	3.24	11.40	36.10	22.30	0.41	2.39	0.29	0.05	0.04	[168]

FA—fatty acid, RA—rumenic acid, LA—linoleic acid, ALA—α-linolenic acid, EPA—eicosapentaenoic acid, DHA—docosahexaenoic acid, NA—data not available, EO—essential oil.

**Table 3:** Effect of alternative and unconventional feedstuffs used in dairy diets on selected sums of milk fatty acids (g/100 g FA).

	Sums of FA (g/100g)						
Feed Supplement	n6	n3	n6/n3	SFA	MUFA	PUFA	Source
**Macroalgae**							
*Ascophyllum nodosum*	1.85	0.59	3.14	74.93	19.19	3.35	[188]
**Microalgae**							
*Aurantiochytrium limacinum*	2.54	0.90	2.82	67.82	27.86	4.32	[193]
*Aurantiochytrium limacinum*	1.96	0.50	3.92	72.62	24.20	3.04	[186]
*Schizochytrium limacinum*	NA	NA	6.78	54.92	36.44	6.05	[77]
*Schizochytrium* spp.	4.56	0.83	5.49	70.95	23.54	5.51	[85]
*Schizochytrium* spp.	1.38	1.54	0.89	53.90	33.60	4.70	[187]
*Schizochytrium* spp.	NA	NA	NA	NA	NA	4.61	[194]
*Schizochytrium* spp.	5.92	1.69	3.50	70.60	19.60	9.82	[86]
*Spirulina platensis*	2.20	0.56	4.06	70.30	25.50	3.30	[67]
*Spirulina platensis*	1.85	0.56	3.36	74.34	18.61	3.25	[8]
*Chlorella vulgaris*	3.68	0.71	5.43	68.20	25.50	4.85	[67]
*Chlorella vulgaris + Nannochloropsis gaditana*	2.70	0.83	3.22	68.70	25.90	4.13	[67]
Rumen-protected macroalgae (Algamac-3050; Aquafauna Bio-Marine Inc., Hawthorne, CA, USA)	3.67	0.94	3.99	55.63	29.31	4.61	[185]
Rumen-protected microalgae DHA-rich microalgae protected with the inert fat (not specified)	4.56	0.83	5.49	61.90	31.21	5.45	[79]
**By-products**							
Okara meal	2.07	0.63	3.26	72.69	22.11	5.19	[99]
Camelina expeller	2.00	0.48	0.45	56.05	37.92	6.02	[117]
Camelina expeller	NA	1.40	NA	62.60	29.70	7.27	[119]
Camelina seeds whole	2.25	0.62	0.39	62.89	32.48	4.63	[117]
**Tanniferous plants**							
Oak tannin	NA	NA	NA	64.23	30.46	5.31	[139]
Hazel	1.62	0.99	1.64	71.80	23.10	3.76	[183]
Silver birch	1.67	0.95	1.77	71.80	23.20	3.62	
Black current	1.76	1.12	1.48	68.00	26.40	4.17	
Grape wine	1.73	1.08	1.61	70.50	24.10	3.96	
Wood avens	1.86	1.14	1.64	70.10	24.40	4.15	
Rosebay willow	1.85	1.17	1.59	70.40	24.10	4.11	
**Herbs and spices**							
Hops	NA	NA	NA	64.09	30.71	5.20	[139]
Oregano low in EO (0.12%) (*Origanum vulgare* ssp. *vulgare*)	NA	NA	NA	78.60	18.34	3.06	[148]
Oregano high in EO (4.21%) (*Origanum vulgare* ssp. *hirtum*)	NA	NA	NA	77.04	19.76	3.20	[148]
**Others**							
Cactus cladode silage	3.14	0.46	6.83	53.40	36.40	6.70	[160]
Blue lupin	5.22	0.47	11.20	67.90	28.50	3.56	[168]

SFA—saturated fatty acid, MUFA—monounsaturated fatty acid, PUFA—polyunsaturated fatty acid, NA—data not available, EO—essential oil.

**Table 4:** Main effects of alternative and unconventional feedstuffs used in dairy diets on indices characterizing health and technological properties of milk fat.

	Indices						
Feed Supplement	AI	HPI	S/P	DI	SI	Main Effect	Source
**Macroalgae**							
*Ascophyllum nodosum*	4.12	0.25	2.53	57.38 (C18)	NA	minor changes in isoC14:0, C15:0, C14:1t4, C18:1c11, C20:1c11, C22:4c7c10c13c16	[188]
**Microalgae**							
*Aurantiochytrium limacinum*	2.54	0.40	1.60	7.78	0.53	- decrease in SFA, C16:0, C20:3n3, n6/n3 - increase in PUFA, n-3 FA, C18:1 t11, C18:2c9t11, C20:5n-3; C22:6n-3	[193]
*Aurantiochytrium limacinum*	3.31	0.31	2.03	7.39	0.58		[186]
*Schizochytrium limacinum*	1.50	0.70	1.03	67.44 (C18)	0.99	- decrease in C12:0–C16:0, C18:0, and n6/n3 - increase in C18:1t, CLA, C18:3n3, C20:5n3, C22:6n3	[77]
*Schizochytrium* spp.	2.88	0.35	1.67	8.51	0.60		[85]
*Schizochytrium* spp.	1.69	0.62	1.03	6.43	0.64		[187]
*Schizochytrium* spp.	NA	NA	NA	8.15	0.25		[194]
*Schizochytrium* spp.	3.28	0.33	1.68	8.44	NA		[86]
*Spirulina platensis*	2.80	0.36	1.81	7.30	0.64	- incease in C18:3n3, C18:3c6c9c12, and PUFA	[67]
*Spirulina platensis*	4.67	0.21	2.78	7.47	0.41		[8]
*Chlorella vulgaris*	2.48	0.41	1.66	6.56	0.68	- increase in C20:5n3, C18:3n3, C18:3c6c9c12, and PUFA	[67]
*Chlorella vulgaris +* *Nannochloropsis gaditana*	2.60	0.39	1.69	7.28	0.65	- increase in C20:5n3, C18:3n3, C18:3c6c9c12, and PUFA - decrease in n6/n3	[67]
Rumen-protected macroalgae (Algamac-3050; Aquafauna Bio-Marine Inc., Hawthorne, CA, USA)	1.90	0.52	1.32	10.43	0.72	- decrease in C16:0 and SFA - increase in CLA, n3 LC-PUFA, trans 18:1, C18:3n3, C22:6n3	[185]
Rumen-protected microalgae-DHA-rich microalgae protected with the inert fat (not specified)	NA	NA	NA	NA	NA		[79]
**By-products**							
Okara meal	3.27	0.34	2.02	6.25	0.48	- increase in C18:1t10, C18:1t11, C18:2c9,t11, n-6 FAs, and total n3 FAs - decrease in total odd-chain FAs and de novo FAs	[99]
Camelina expeller	1.88	0.58	0.99	12.16	0.50	- decrease in C18:0, C18:1c9, and several n6 FAs - increase in n9 MUFAs (except C18:1c9) and long-chain n6 FAs (20:2c11c14 and C22:2c13c16)	[117]
Camelina expeller	NA	0.48	1.25	7.68	NA		[119]
Camelina seeds whole	2.26	0.46	1.29	9.00	0.47	- decrease in C18:0, 18:1c9, and several n3 FAs - increase in several long-chain SFAs and MUFAs (≥20 C), and long-chain n3 FAs (20:3c11c14c17 and 22:3c13c16c19)	[117]
**Tanniferous plants**							
Oak tannin extracts	2.55	0.46	1.47	7.87	0.88	- increase in C18:3n3 - decreased C18:2n6:C18:3n3 ratio	[139]
Hazel	4.04	0.26	2.20	38.64 (RA)	NA	- increase in C16:0 - decrease in C18:2c9t11, C15.0, C15:1, C16:1, iso C17, PUFAs	[183]
Silver birch	3.97	0.26	2.17	38.33 (RA)	NA	- decrease in C18:2c9t11, C15:1, C16:1, iso C17, PUFAs	[183]
Black current	3.30	0.32	1.84	35.90 (RA)	NA	- increase in C18:1c11 - decrease in C15.0, C15:1, C16:1, iso C17	[183]
Grape wine	3.76	0.28	2.03	50.00 (RA)	NA	- decrease in C18:2c9t11, C15.0, C15:1, C16:1, iso C17	[183]
Wood avens	3.70	0.28	2.00	38.57 (RA)	NA	- decrease in C18:2c9t11, C15.0, C15:1, C16:1, iso C17	[183]
Rosebay willow	3.73	0.28	2.04	36.57 (RA)	NA	- decrease in C18:2c9t11	[183]
**Herbs and spices**							
Hops	2.55	0.46	1.47	8.08	0.86	- no effect on milk FAs	[139]
Oregano low in EO (0.12%) (*Origanum vulgare* ssp. *vulgare*)	2.11	0.55	2.09	70.35 (C18)	0.37	- increase in C18:3n3	[148]
Oregano high in EO (4.21%) (*Origanum vulgare* ssp. *hirtum*)	1.87	0.62	1.90	69.16 (C18)	0.41	- no effect on FAs	[148]
**Others**							
Cactus cladode silage	1.39	0.78	1.04	6.51	0.94	- increase in 18:1 t11, 18:2n6, 18:3n3, C18:2c9t11 - decrease in C18:0 and 18:1c9	[160]
Blue lupin	2.66	0.38	1.80	9.88	0.62	- increase in UFAs and MUFAs - decrease in SFAs and n6/n3	[168]

FA—fatty acid, SFA—saturated fatty acid, MUFA—monounsaturated fatty acid, UFA—unsaturated fatty acid, PUFA—polyunsaturated fatty acid, AI—atherogenic index, HPI—health-promoting index, DI—desaturation index calculated from C14 FA unless otherwise noted (see Section 2 for the formulas), SI—spreadability index, NA—data not available, EO—essential oil.

## 5. Conclusions

The demand for high quality milk is constantly growing. Studies in the area of milk fat have focused mainly on ways to increase the content of polyunsaturated and n3 fatty acids and to improve the n6/n3 ratio. Aside from the thoroughly-studied dietary factors and common feedstuffs, there are also some alternative or unconventional feedstuffs that are often used for different purposes (e.g., for the reduction of methane emissions), which may modify the FA profile of milk fat. However, the effects of various feedstuffs on milk fatty acid profiles are difficult to compare due to the large spectrum of individual FAs that should be taken into consideration and due to differences in the applied dietary factors, such as the kind of feedstuff and its proportion/amount in the diet, the composition of the basal diet, as well as the nature of the supplements and the length of supplementation. Thus, using indices characterizing the health properties of milk fat can be helpful.

## Data Availability

No new data were created or analyzed in this study. Data sharing is not applicable to this article.
